# *Malva sylvestris* Flower Extract Exhibits Antineoplastic Potential Against Human Colon Cancer Cell Lines and Induces *CDK2* Transcript Instability via Plant miR160-5p

**DOI:** 10.3390/nu18030495

**Published:** 2026-02-02

**Authors:** Valentina Villani, Angelo Gismondi

**Affiliations:** 1 Laboratory of Botany, Department Biology, University of Rome Tor Vergata, Via Della Ricerca Scientifica 1, 00133 Rome, Italy; 2 PhD Programme in Molecular and Cellular Biology, Department of Biology, University of Rome Tor Vergata, 00133 Rome, Italy

**Keywords:** common mallow, plant microRNA, cross-kingdom regulation, antiproliferative effect, antimetastatic power, antiinvasive property, cyclin-dependent kinase 2, senescence, differentiation

## Abstract

**Background:** *Malva sylvestris* (the common mallow) is an herbaceous species widely used in ethnobotanical practices to treat gastrointestinal, hepatic and urinary inflammation. **Objectives:** Despite these beneficial effects on human health, the antineoplastic potential of this plant has not yet been fully explored. Thus, in the present study, two human colon cancer cell lines (i.e., HCT-116 and Caco-2) were treated with an extract obtained from *M. sylvestris* flowers (MFE), whose composition in terms of phytochemicals and microRNAs has been recently published by our research group, to explore its potential bioactivity. **Methods/Results:** MTT and Trypan blue assays demonstrated that MFE reduced tumour cell growth without causing significant cytotoxicity or apoptosis. Following the diphenylboric acid 2-aminoethyl ester-induced fluorescence of some plant metabolites, microscopy analysis proved that MFE components crossed the cell membranes, accumulating into nuclei. Wound assay and transwell tests documented that MFE was also able to reduce cell motility and invasiveness. In both cell lines qPCR experiments demonstrated that MFE caused the over-expression of factors, like *VIMENTIN* and *E-CADHERIN*, which negatively influence epithelial–mesenchymal transition in colon cancers. However, the effects of MFE appeared to be time-, dose- and cell type-dependent. In fact, the treatment induced senescence in P53-null Caco-2 cells (i.e., ROS, β-galactosidase and P21^WAF1/Cip1^) and a premise of differentiation (i.e., P27^Kip1^) in P53-wild-type HCT-116 cells, also via the CDK2/c-MYC/AKT axis, justifying its antiproliferative property. In parallel, the transfection of tumour cells with pure synthetic miR160b-5p—a microRNA identified in *M. sylvestris* flowers and predicted to target the human *CDK2* transcript—resulted in gene silencing, thereby suggesting its central role in mediating the cross-kingdom effects of MFE on the investigated cancer models. **Conclusions:** Overall, these findings open new perspectives on the common mallow as a source of potential antimetastatic compounds and on the possible use of its plant microRNAs in the development of gene therapies.

## 1. Introduction

Plants represent complex matrices of biologically active compounds, such as phenolics, alkaloids and terpenoids. The effect of these substances within heterologous biological systems—specifically animal and human models—constitutes the pharmacological foundation of phytomedicine. Indeed, since time immemorial, plant extracts have been used for therapeutic purposes due to their antioxidant, antimicrobial, anti-inflammatory and immunomodulatory properties. Despite their historical use, botanical derivatives continue to undergo rigorous phytochemical analysis to isolate novel bioactive scaffolds with promising pharmaceutical applications [1].

Simple phenols and flavonoids, which are characterized by high selectivity and low systemic toxicity, have emerged as promising lead compounds for the development of novel anticancer therapies. These phytochemicals have primarily been reported to exert their antineoplastic potential by modulating the redox balance within the tumour microenvironment. Specifically, they act as pro-oxidants in cancer cells, leading to an overproduction of reactive oxygen species (ROS) that triggers programmed cell death pathways, including apoptosis and autophagy. Furthermore, these metabolites are known to induce cell cycle arrest and to modulate intracellular signalling cascades (e.g., PI3K/Akt and MAPK), thus suppressing tumour progression [2,3]. Interestingly, in other cases, these substances have demonstrated significant antineoplastic activity by promoting terminal differentiation or triggering cellular senescence by upregulating specific markers, such as p27^Kip1^ and β-galactosidase [4,5,6,7]. However, in this context, one of the most debated issues is linked to the intrinsic low bioavailability of phytochemicals, which, together with the lack of univocal information about their molecular mechanism of action, hinders the translation of the laboratory evidence in human clinical trials and therapies [8,9].

Recently, it has been proposed that medicinal and food plants may modulate their consumers’ health not only through phytochemicals but also via microRNAs (miRNAs, miRs). Plant miRNAs, which are short non-coding RNAs, have been documented to be very stable, resistant to extracellular environments, absorbable in mammalian cells via exosomes or by RNA transporter membrane proteins and able to carry out cross-kingdom regulation of mammalian gene expression. In fact, by the RNA-induced silencing complex (RISC), they interact with specific mRNA targets, inducing their degradation or translation inhibition [10,11,12]. All this evidence is expanding the perspectives of the pharmacognosy, even suggesting plant miRNAs-based gene therapy approaches [13,14,15].

*Malva sylvestris* L. (the common mallow) is among the numerous species of ethnobotanical interest. To date, several studies have demonstrated the antiphlogistic, antiulcerogenic, antibacterial and laxative efficiency of this medicinal plant, promoting the application of its extracts as ingredients for modern pharmaceutical preparations, cosmetics and personal care products and underlining the necessity to extend the research towards the clinical and toxicological aspects of its use. In fact, the literature data indicate that *M. sylvestris* is safe at low concentrations, although further research is required to evaluate potential negative consequences associated with its high dosages and prolonged consumption [16,17,18,19]. Since scholars have mainly focused their studies on the leaves of *M. sylvestris*, recently, our research group has investigated the flower tissue. By chromatographic and spectrophotometric analyses, we have shown that it is rich in phytochemicals, such as phenols (e.g., 3-hydroxytyrosol and vanillic acid), flavonoids (e.g., epicatechin and genistein) and anthocyanins (e.g., malvidine-3-glucoside and petunidin-3-glucoside), and that it contains 23 miRNAs, although just 8 of them are typifying this plant organ (i.e., miR160b-5p, miR396c-3p, miR159c-3p, miR6300, miR3954b-5p, miR395c-3p, miR166g-3p, and miR164d-5p) [20]. Taken together, these findings suggest that the common mallow flower is a promising novel plant matrix for investigating potential biological activities.

In this context, it is important to underline that, to the best of our knowledge, only a couple of works in the literature have documented the antineoplastic potential of *M. sylvestris*. One was that by Alesiani et al. [21], who have suggested the possibility that hydroalcoholic extract (70% methanol in water; *v*/*v*) from *M. sylvestris* leaves could induce cytotoxicity against murine (B16) and human (A375) melanoma cells, in line with the evidence documented by Rayssan and Shawkat [22] on melanoma and lymphoma model systems. By contrast, Conforti et al. [23] have found that another methanolic preparation from a similar plant material did not exert any significant effect on MCF-7 (human breast cancer cells), LNCaP (human prostate cancer cells), ACHN (human renal cell adenocarcinoma), and C32 (human amelanotic melanoma cells) lines. Thus, to fill this gap in the scientific knowledge, the aim of the present contribution was to investigate the antiproliferative, antimetastatic and antiinvasive power of *M. sylvestris* flower extract against two human colon cancer cell lines (i.e., HCT-116 and Caco-2) and to link its antitumor potential to the bioactivity of miR160b-5p, one of the plant miRNAs recently identified in the common mallow flower miRNome by our research group [20].

## 2. Materials and Methods

### 2.1. Plant Material and Extraction Procedure

*M. sylvestris* flowers were collected in June 2019 from plants grown in the Botanical Gardens of Rome Tor Vergata. The plant material was powdered, with mortar and pestle, in the presence of liquid nitrogen, and stored at −80 °C until the analysis. Thus, 150 mg of flower powder were mixed with 1 mL of ethanol at 50% and left to macerate in agitation for 24 h at room temperature in the dark. After 20 min of centrifugation at 11,000× *g*, the supernatant was filtered with a 0.22 µm sterile membrane filter (Sigma-Aldrich, Milan, Italy) and totally desiccated at 30 °C by a vacuum dry evaporating system (Concentrator Plus, Eppendorf, Hamburg, Germany), to generate a pellet containing common mallow molecules.

### 2.2. Cell Cultures and Plant Treatments

Human colon cancer (HCT-116) and human colorectal adenocarcinoma (Caco-2; undifferentiated) cell lines were maintained and propagated under standard conditions [24]. Specifically, cells were cultured in Dulbecco’s Modified Eagle’s Medium (DMEM), supplemented with 10% fetal bovine serum (FBS), 200 mM glutamine, 100 U/mL penicillin and 0.1 mg/mL streptomycin, and maintained in a humidified incubator at 37 °C with 5% CO_2_. Non-tumour immortalized human colon epithelial cells (HCEC-1CT) were cultured in ColoUp medium (Evercyte GmbH, Vienna, Austria), supplemented with 100 U/mL penicillin and 0.1 mg/mL streptomycin, and maintained in a humidified incubator at 37 °C in 5% CO_2_. All cell types were harvested twice a week and used at about 80% confluence. For cell experiments, the plant pellet, obtained as reported above and equivalent to the extract of 150 mg of flower powder, was resuspended in 0.5 mL of DMEM or ColoUp (according to the cell line used), in order to reach the final concentration of phytochemicals contained in 300 mg of plant material per mL of solvent (that is, 30% suspension; henceforth, mallow flower extract—MFE), centrifuged at maximum speed for 5 min, filtered again (0.22 µm) and collected in a new tube, to be used for the treatments. To carry out the treatments, cells were exposed, for 24 h or 48 h, to different doses of MFE (i.e., the content of plant molecules isolated from 0.9, 6 and 15 mg of original material per mL of culture medium). The control (CNT) samples consisted of cells treated with pure DMEM or ColoUp for the same time as and volume of the highest treatments of the series. For transfection assays, cells were plated (1 × 10^5^) for 24 h and then transfected for another 48 h with the synthetic 3′/2C-*O*-methylated miR160b-5p (5′-UGCCUGGCUCCCUGUAUGCCG-3′*), or with a synthetic 3′/2C-*O*-methylated scrambled miRNA used as a system control (henceforth SiR; 5′-GACACGCGACUUGUACCAC-3′*), at the final concentration of 98 nM. All transfections were performed using lipofectamine reagent (X-treme GENE siRNA Transfection Reagent, Sigma Aldrich, Milan, Italy), according to the manufacturer’s instructions.

### 2.3. Trypan Blue Exclusion Test and Cell Growth

The cytotoxicity of the plant extract was assessed by a Trypan blue exclusion test. Briefly, the number of alive and dead (blue-coloured) cells was counted after 24 and 48 h of treatment for 1 min with 1% *w*/*v* Trypan blue dye (Sigma-Aldrich, Milan, Italy), using a Neubauer microchamber (Brand GmbH, Wertheim, Germany) under a light microscope. In parallel, cell growth was evaluated by the application of the 3-(4,5-dimethyl-thiazol-2-yl)-2,5-diphenyltetrazolium bromide (MTT) kit (Sigma-Aldrich, Milan, Italy), following the manufacturer’s guidelines. Results were expressed as the number of cells for the Trypan blue assay and as a percentage variation in the growth with respect to the control taken as a unit (100%) in the MTT test.

### 2.4. Fluorescence Microscopy

Cells were seeded on a glass cover slip in 6-well plates and incubated with fresh complete medium for 24 h prior to treatment with the plant extract and then for another 48 h. Then, they were fixed with 4% paraformaldehyde for 20 min in the dark at room temperature. Nuclei were stained with DAPI (1 μg/mL in PBS, phosphate-buffered saline), and their signal was monitored under UV-light, while some phytochemicals, like flavonoids, were marked using diphenylboric acid 2-aminoethyl ester (DPBA, 2.5 mg/mL for 10 min), a fluorescent probe, detectable after excitation at 458 nm, following the principles and methods in the literature [25,26,27], by using an Olympus (Evident, Tokyo, Japan) FV4000 Confocal Laser Scanning Microscope.

### 2.5. Oxidative Stress Evaluation

After exposure to the plant extract, cells were incubated with 2′,7′-dichlorodihydrofluorescein diacetate (DCFH-DA; 20 μM; Sigma-Aldrich, Milan, Italy) for 30 min at 37 °C, in the dark. Then, samples were washed three times with PBS 1X and collected in cytofluorimetric tubes. Fluorescence was measured on 10,000 cell events per sample, using an appropriate filter (FITC, FL1-H) and a FACSCalibur instrument (Beckton and Dickinson, San José, CA, USA). Basal controls were carried out by exposing cells only to DCF-DA (stained CNT). Negative controls were performed by treating cells with pure culture medium (unstained CNT) and with the plant extract but without DCFH-DA, whereas positive ones were carried out by incubating cells with H_2_O_2_ (5 mM for 4 h) before the treatment with the probe. Results were expressed as percentage variation of fluorescence with respect to the CNTsample, taken as unit (100%).

### 2.6. Wound Assay and Migration and Invasion Tests

For wound assay, cells were seeded in 6-well plates (2 × 10^5^ cells/well) and grown for 4 days to reach about 80% of confluence. Then, artificial wounds were generated on the cell monolayer using a 200 µL micropipette tip. Images were acquired immediately (0 h) and after 24 and 48 h of treatment with the plant extract by an AxioCam MRc camera associated with an AxioVert25 microscope (Zeiss, Oberkochen, Germany). AxioVision Rel 4.6 program and ImageJ (version 1.48v; Bethesda (Rockville, MD, USA), NIH, USA) software were used to trace and calculate the wound area, which was expressed in percentage largeness with respect to the initial dimension (taken as unit, 100) as a function of time. In parallel, 2 × 10^5^ cells were plated in each lodge of the 6-well plates and grown for 2 days under normal conditions. Then, the medium was removed, and cells were treated for 48 h with the plant extract in the presence of serum-free culture medium. After that, to evaluate the migration power, cells were loaded on transwell chambers (Corning, Sigma-Aldrich, Milan, Italy) separated by membranes showing 8 µm-apertures. The invasiveness potential was estimated by using the same system but applying a layer of Matrigel (Sigma-Aldrich, Milan, Italy), by dropping 80 μL of matrix on the membranes and allowing its polymerization at 37 °C for 1 h. In the chamber containing the cells, serum-free culture medium was added, while on the other side, medium containing 10% FBS was inserted. The transwell chambers were incubated for 48 h at 37 °C. Thus, the membranes were collected, fixed in 4% paraformaldehyde for 15 min at room temperature and stained with 0.1% crystal violet. Cells on the upper surface of the filter were removed by wiping with cotton, while those able to move and present on the lower surface were detected by staining with Coomassie brilliant blue and counted using an optic microscope. For the analysis, 10 fields of each membrane were randomly selected and photographed.

### 2.7. Cell Cycle and Senescence Analysis

Cells were washed twice with PBS 1X and fixed in a cold methanol:acetone (4:1, *v*/*v*) solution at 4 °C for 30 min. Then, samples were incubated with RNase A (100 μg/μL) for 20 min and stained with propidium iodide (1 mg/mL) for 20 min. Finally, they were subjected to cytofluorimetric analysis (10,000 events per sample) by a FACSCalibur instrument (FL-2; Beckton and Dickinson, San José, CA, USA) associated with CellQuest software (v.5.1). Senescent cells were analyzed using a specific staining kit (Senescence Cells Histochemical Staining Kit; Sigma-Aldrich Co.), according to the manufacturer’s guidelines. The kit identifies senescent cells by detecting senescence-associated beta-galactosidase activity on a chromogenic substrate, which (once cleaved) undergoes oxidation and dimerization, forming a stable dark green precipitate within the cytoplasm. Senescent cells, marked in dark green, were counted by light microscopy (20X, Nikon ECLIPSE E100, Amstelveen, The Netherlands) from optic fields (10 per sample) captured randomly by an AxioCam MRc camera associated with an AxioVert25 system.

### 2.8. Quantitative Real-Time PCR (RT-qPCR)

To carry out the gene expression analysis, total RNA was extracted from cells using the Pure Link RNA Mini Kit (Ambion, Life Technologies, Carlsbad, CA, USA), according to the manufacturer’s protocol. The RNA concentration and purity were determined by a Nanodrop ND1000 spectrophotometer (NanoDrop Technologies, Wilmington, DE, USA). cDNA was synthesized starting from 2.5 µg of extracted RNA. The RNA was incubated for 2 min at 65 °C, with 0.4 mM of each dNTP (Euroclone, Milan, Italy). Then, 40 U/µL of RNA inhibitor (RNasin; Promega, Madison, WI, USA), 0.5 µg of random hexamer primers (from a stock solution of 0.5 µg/µL; Invitrogen, Monza, Italy), 200 U/µL of Moloney murine leukemia virus reverse transcriptase (Promega, Milan, Italy; cat. num.: M1701), 1X of enzyme buffer and dithiothreitol (final concentration 10 mM) were added; finally, water was used to reach a final volume of 25 µL. The mix was incubated for 90 min at 37 °C and then for 5 min at 85 °C to inactivate the reaction. Real-time PCR reactions were performed in a 20 µL reaction mixture composed of 20 ng of cDNA, 5 µM of each primer (Supplemental Material—Table S1) and 50% SYBR green (Kapa SYBR Fast qPCR kit; Kapa Biosystems, Woburn, MA, USA; cat. num.: KK4607). Amplifications were performed in a IQ5 thermocycler (Bio-Rad) with the following method: (a) initial denaturation at 95 °C for 4 min; (b) 40 cycles of denaturation at 95 °C for 20 s (sec), primer annealing at 60 °C (for all genes) for 30 s, and extension at 72 °C for 30 s; (c) production of a disassociation curve, from 50 to 90 °C (rate: 0.5 °C every 5 s). The amount of mRNA for each gene was quantified using the 2^−ΔΔCt^ formula, where the threshold cycle (Ct) of the target gene detected in the treated sample was normalized for the internal reference gene (β-ACTIN, ΔCt; also, GAPDH was used as an internal loading control and results were comparable) and for the respective value observed in the control sample (ΔΔCt), considered as unit. To perform miRNA expression analysis, total miRNAs were isolated from human cells using the mirPremier microRNA Isolation Kit (Sigma-Aldrich), retrotranscribed by the miRCURY LNA Universal RT microRNA PCR-Synthesis Kit II (EXIQON, Vedbæk, Denmark), and quantified by applying the Real Time qPCR method, as described in Gismondi et al. [28] and Gismondi et al. [29]. The primers used in qPCR amplifications were specifically designed and synthesized by EXIQON (microRNA LNA PCR primer sets, EXIQON), according to the miRNA sequences obtained from miRBase (http://www.mirbase.org/). The estimation of miR160b-5p content was carried out by an absolute quantitation method, that is, using a standard curve adequately created by analyzing increasing concentrations of the pure synthetic miR160b-5p, while U6 small RNA was used as an internal loading control [10].

### 2.9. Immunoblotting

Cells were washed with PBS 1X, lysed for 20 min at 4 °C in high salt buffer (2 mM CaCl_2_, 350 mM KCl, 50 mM Tris HCl pH 7.4 and 1 mM MgCl_2_) containing 1% protease inhibitor cocktail and 1% NP40, and centrifuged at 12,000× *g* for 30 min at 4 °C. Total protein concentration was estimated by the Bradford method. Fifty μg of proteins were boiled at 95 °C in Laemmli-SDS loading buffer for 5 min, separated by SDS-PAGE (12%) and transferred onto a nitrocellulose membrane. After blocking with 5% bovine serum albumine for 1 h, the membranes were incubated with primary antibody for 3 h and then with peroxidase-conjugated secondary antibody for 1 h. Their signal was detected by using a chemiluminescent kit (Western Blotting Luminol Reagent; Santa Cruz Biotechnology, Dallas, TX, USA) by a VersaDoc Imaging System associated with a Quantity One software (v. 4.6.7, Bio-Rad, Hercules, CA, USA). Results were expressed as relative variation with respect to the CNT sample, taken as unit. The following primary antibodies were purchased from Santa Cruz Biotechnology (Dallas, TX, USA): mouse monoclonal β-ACTIN (sc-58673), mouse monoclonal P53 (sc-1314), rabbit polyclonal P27^Kip1^ (sc-776), mouse monoclonal PARP-1 (sc-375150), mouse monoclonal Caspase-3 (CASP-3) (sc-56046), rabbit monoclonal BCL-2 (sc-7382), rabbit monoclonal Bax (sc-70407) and rabbit polyclonal total AKT (sc-1618). Rabbit polyclonal phoshpo-AKT (Ser473) (cs-4060), mouse monoclonal cleaved CASPASE-3 (cCASP-3) (cs-94530), rabbit monoclonal phospho-CDK2 (Thr160) (cs-2561), mouse monoclonal phosho-c-MYC (Ser62) (cs-13748) and mouse monoclonal P21^WAF1/Cip1^ (cs-2946) were purchased from Cell Signaling Technology (Beverly, MA, USA). The horseradish peroxidase-conjugated anti-mouse (AP308P), anti-goat (G5018) and anti-rabbit (A0545) used as secondary antibodies were derived from Merck (Milan, Italy).

### 2.10. Statistical Analysis

All results were expressed as mean value ± standard deviation (s.d.) of 3 different measurements (technical replicates) each obtained from 3 independent experiments (biological replicates). Statistical significance was evaluated, through the Excel software, by the one-way ANOVA test and the post hoc Lowest Standard Deviations test. Only results associated with a *p*-values <0.05 were considered statistically significant (*p*-values: * < 0.05; ** < 0.01; *** < 0.001).

## 3. Results

**Figure 1:**
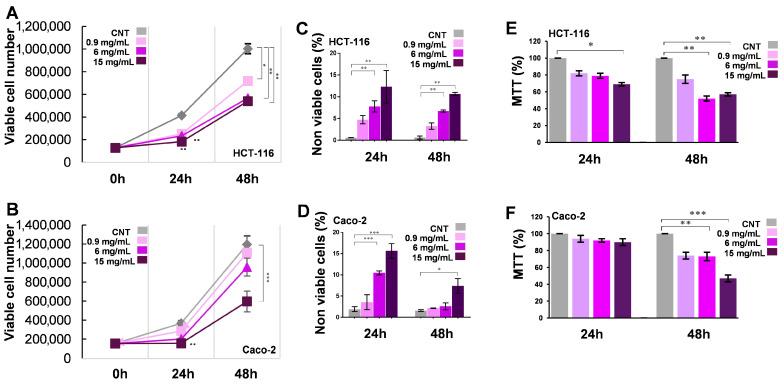
Antiproliferative effects of MFE. (**A**,**B**) Proliferation curves of HCT-116 (panel (**A**)) and Caco-2 (panel (**B**)) cells generated by counting the number of live cells (i.e., unstained by Trypan blue reagent) after treatments with different concentrations of MFE for 0, 24 and 48 h. (**C**,**D**) Percentage of dead HCT-116 (panel (**C**)) and Caco-2 (panel (**D**)) cells (i.e., stained by Trypan blue reagent) after treatments with different concentrations of MFE for 24 and 48 h. (**E**,**F**) Percentage of cell growth, compared to the CNT taken as unit (100%), of HCT-116 (panel (**E**)) and Caco-2 (panel (**F**)) cells measured by an MTT assay after treatments with different concentrations of MFE for 24 and 48 h. In all panels, data were indicated as mean ± s.d. of three independent experiments (* *p* < 0.05 vs. control; ** *p* < 0.01; *** *p* < 0.001).

**MFE affects the HCT-116 and Caco-2 proliferation rate.** The biological effect of the mallow flower extract (MFE) was evaluated on the HCT-116 and Caco-2 human colorectal tumoural cell lines. First, the antiproliferative properties of three different concentrations of MFE (phytochemicals equivalent to 0.9, 6 and 15 mg of plant material per mL of culture medium) were estimated using a Trypan blue exclusion test. HCT-116 and Caco-2 cell proliferation curves were generated by counting live cells (Figure 1A,B) after 24 and 48 h of treatment. The cell number for both lines was significantly affected by MFE in a dose- and time-dependent manner, although the Caco-2 cells seemed to be less sensitive to the plant extract. The strongest effects were observed after 48 h of incubation with the highest doses of MFE (15 mg/mL), reaching a decrease in proliferation equal to 46% for HCT-116 and 50% for Caco-2, compared to the respective controls (CNTs). In parallel, dead cells, clearly stained in dark blue by the reagent, were also counted in each sample (Figure 1C,D) to measure the cytotoxicity level of MFE on the two cell systems. The percentage of dead cells was always less than 20% in both lines. However, a slight increase in toxicity could be detected in all treated samples, especially after 24 h; in fact, after an initial acute response, the number of dead cells decreased at 48 h, suggesting a minimal cytotoxic potential for the *M. sylvestris* extract on these two tumour cell lines. To exclude the possibility that MFE could be toxic for non-tumor systems, HCEC-1CT cells, an immortalised human colon epithelial cell line, were also exposed to the plant extract for the same times and concentrations. No significant change in the number of live or dead cells was observed in this case after Trypan blue staining, suggesting that the tested mallow extract was safe for non-cancerous cells (Supplemental Material—Table S2).

To confirm the previous observation, an MTT assay was also performed for the same experimental points reported above (Figure 1E,F). The results were in line with those obtained by the Trypan blue assay: in particular, 15 mg/mL of MFE reduced HCT-116 and Caco-2 cell growth by 44% and 53%, respectively, after 48 h, with respect to the corresponding CNTs.

**Figure 2:**
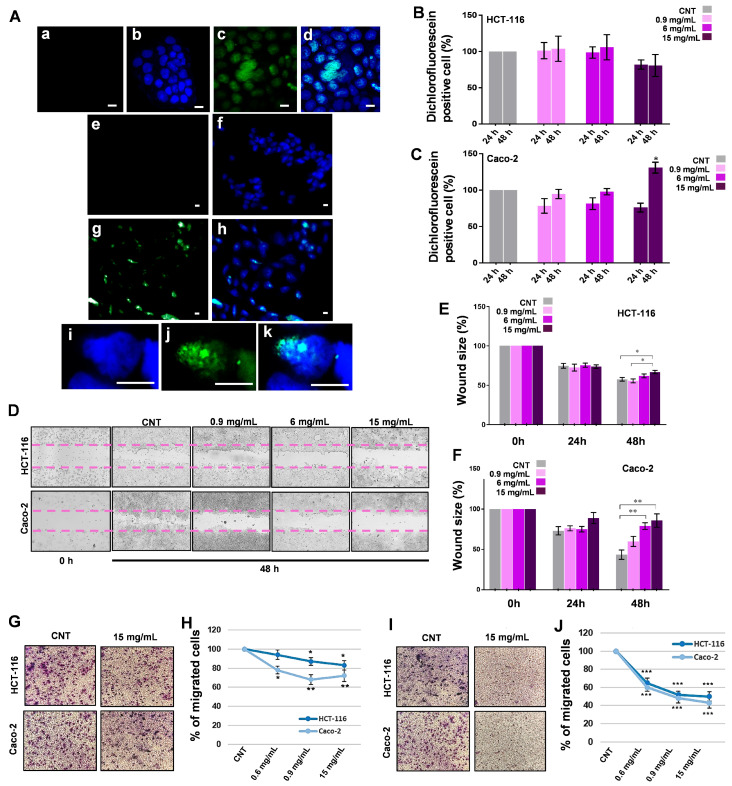
Fluorescence analyses and antimetastatic effects of MFE. (**A**) Representative images of HCT-116 (subpanels (**Aa**–**Ad**,**Ai**–**Ak**)) and Caco-2 (subpanels (**Ae**–**Ah**)) cells captured by fluorescent microscopy. HCT-116 CNT cells correspond to subpanels (**Aa**,**Ab**), while subpanels (**Ae**,**Af**) to those of Caco-2. HCT-116 exposed to the highest dose of MFE for 48 h were shown in subpanels (**Ac**,**Ad**,**Ai**,**Aj**,**Ak**), whereas treated Caco-2 cells are reported in the subpanels (**Ag**,**Ah**). Subpanels (**Aa**,**Ac**,**Ae**,**Ag**,**Aj**) report the localisation of phytochemicals (green signal), while subpanels (**Ab**,**Ad**,**Af**,**Ah**,**Ak**) are the merge between the previous fluorescence images and their respective visualization under UV light used to detect the nuclei staining with DAPI reagent (blue signal). Subpanels (**Ai**–**Ak**) represent the magnification of a single cell. In each subpanel, the white bar indicates 10 µm. (**B**,**C**) Intracellular ROS level quantified in HCT-116 cells (panel (**B**)) and Caco-2 (panel (**C**)) cells treated with different doses of MFE for 24 and 48 h by DCFH-DA fluorescent assay. ROS concentration was reported as a percentage compared to the CNT. (**D**–**F**) Wound healing assay carried out on HCT-116 and Caco-2 cells exposed to different doses of MFE for 24 and 48 h. Representative microphotographs were taken by light microscopy immediately after the creation of the wound (0 h) and after 48 h of treatment (panel (**D**)). Histograms representing the percentage variations in the sizes of the scratches, with respect to the condition at 0 h taken as a unit (100%), were reported for the HCT-116 (panel (**E**)) and Caco-2 (panel (**F**)) cells. (**G**–**J**) Migration and invasion assays carried out on the HCT-116 and Caco-2 cells exposed to different concentrations of MFE for 48 h. Representative microscopic images of the filters present in the Boyden chamber that were used to perform the experiments, together with the cells fixed on them after staining by Coomassie brilliant blue (panel (**G**) for migration test, panel (**I**) for invasion test). The histograms representing the percentage of the number of cells counted on the filters of the transwell migration system, with respect to the CNT, taken as unit (100%), were reported (panel (**H**) for migration assay, panel (**J**) for invasion assay). In all panels, data were indicated as mean ± s.d. of three independent experiments (* *p* < 0.05 vs. control; ** *p* < 0.01; *** *p* < 0.001).

**Phytochemicals from MFE enter the nuclei and modulate cell redox state.** In order to understand if the phytochemicals present in the plant extract entered into the cells during the treatment or just activated membrane receptors remaining in the culture medium, a fluorescence microscopy analysis was carried out (Figure 2A), using DPBA staining. With respect to the CNTs where only DAPI-stained nuclei were visible (blue spots), MFE-treated Caco-2 and HCT-116 cells clearly showed flavonoid fluorescence (green spots). In particular, in the merged images (e.g., in the magnification reported in Figure 2A—panels Ai, Aj and Ak), it is possible to observe that the two signals overlapped, indicating that MFE phytochemicals not only penetrated within the cells but also localized specifically in the nuclei, perhaps with the aim of modulating cell gene expression.

Since the presence of MFE metabolites in the cells was demonstrated, another important step was to clarify whether the extract was able to induce changes in the cell redox state. For this purpose, the level of intracellular reactive oxygen species (ROS), mainly hydrogen peroxide, was monitored following the fluorescence of the DCF probe by flow cytometry (Figure 2B,C). No significant modification of ROS levels was found in HCT-116 cells, although at the highest concentration, it seemed that reactive species tended to diminish. By contrast, in the Caco-2 cell line, all treatments at 24 h tended to induce a preliminary reduction in ROS, which was then recovered at 48 h. Interestingly, only the highest MFE dose (15 mg/mL) at 48 h was able to induce a moderate (+25.1%) and significant (*p* < 0.05) increase in the content of reactive species, compared to the CNT. As expected, the positive control with hydrogen peroxide caused a strong accumulation of DCF-positive cells (+80.2% in HCT-116; +58,1% in Caco-2, at 48 h).

***M. sylvestris* extract suppresses the migratory and invasive potential of tumour cells.** To investigate the effects of MFE on the migration capability, a typical feature of tumour cells, the confluent monolayers of HCT-116 and Caco-2 were scratched to create a wound. Subsequently, cells were exposed to the plant extract for 24 h and 48 h. For the analysis, the distance between the two edges of the wound at different times was measured to estimate the capability of the cells to repair the cut and proliferate into this space. As shown in Figure 2D, in the CNT samples, the cells migrated rapidly, covering part of the wound area after 48 h. On the contrary, Caco-2 cells exposed to the MFE presented an inhibition of their migratory potential, showing the cut largely uncovered after 48 h of treatment at the highest dose compared to its CNT (−42.2%), while HCT-116 showed just a small effect (−9.1% vs. its control) under the same conditions (Figure 2E,F).

These results were in line with the evidence previously collected about the antiproliferative properties of MFE on the two studied cell lines. However, to deepen this aspect, an invasion test and another migratory assay were carried out by using Boyden chambers with or without the addition of a layer of Matrigel, respectively. After 48 h of treatment with the three different doses of MFE, the motility of both HCT-116 and Caco-2 appeared significantly impaired, as revealed by the number of stained cells that migrated across the filters present in the Boyden chambers (Figure 2G,I) and their quantitation reported in the respective graphs (Figure 2H,J). In particular, the effect of MFE seemed to be dose-dependent and mainly able to block HCT-116 and Caco-2 invasiveness. Indeed, compared to the respective controls, 15 mg/mL of MFE reduced migration capability by 17.1% in HCT-116 and by 29.5% in Caco-2, while the invasion properties were diminished by 48.1% and 56.1%, respectively. All these data demonstrated that the common mallow extract negatively influenced both the invasion and migration power of the studied cell models.

**Figure 3:**
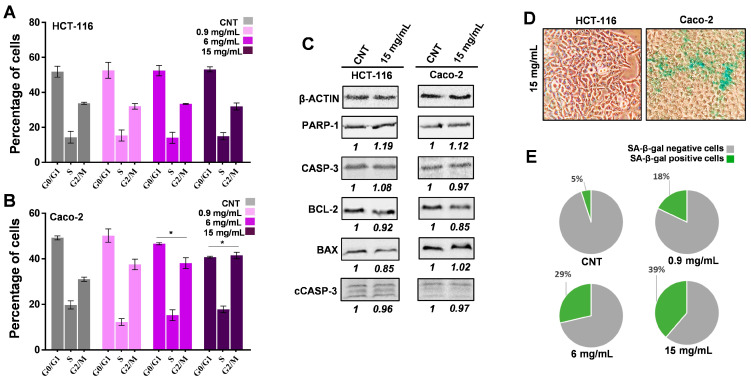
Cell cycle and cell death analysis. (**A**,**B**) Cell cycle analysis of HCT-116 (panel (**A**)) and Caco-2 (panel (**B**)) cells after 48 h of treatments with different doses of MFE. For all samples, the percentage of cells in each cycle phase (G0/G1, S, and G2/M), as measured by flow cytofluorimetry, was reported. (**C**) Representative immunoblots of PARP-1, CASP-3, BCL-2, BAX, cleaved CASP-3 (cCASP-3) and β-ACTIN protein levels in HCT-116 and Caco-2 cells, exposed or not for 48 h to the highest dose of MFE, were shown. Protein expression levels were quantified relative to the control (CNT), which was assigned a value of 1. These values are reported directly beneath the representative images. β-ACTIN served as the loading control to ensure accurate normalization of the results. (**D**) Representative microscopy images of HCT-116 and Caco-2 cells treated for 48 h with the highest dose of MFE and subjected to the senescence kit. Green cells are those in which senescence was triggered. (**E**) Pie charts showing the percentage of senescent events counted in Caco-2 cells exposed for 48 h to the highest dose of MFE. In all panels, data were indicated as mean ± s.d. of three independent experiments (* *p* < 0.05 vs. control).

**MFE Induces Cell Cycle Arrest and Senescence in Caco-2 but Not in HCT-116 Cells.** Taking into account the previous results about the antiproliferative activity promoted by MFE on the two tumour cell lines, a disruption of cell cycle progression was hypothesized. To check this, a cytofluorimetric analysis was performed after 48 h of incubation with *M. sylvestris* extract. As shown in Figure 3A, no treatment resulted in alterations of the cell cycle in HCT-116 cells; by contrast, the highest concentration of MFE (15 mg/mL) significantly induced a reduction in G0/G1 events (−10.43%) and a consequent accumulation of cells in S and G2/M phase (Figure 3B).

To complete this scheme and confirm the inability of MFE to induce cell death, as previously hypothesized, considering that no significant cytotoxicity was detected by Trypan blue assay, the protein content of pro- or anti-apoptotic factors (i.e., PARP-1, CASP-3, cleaved CASP-3, BCL-2 and BAX) was assessed by immunoblotting analysis. As demonstrated by the representative blots and relative densitometric quantitation (Figure 3C), the common mallow extract did not lead to any significant variation in these targets in both cell lines after 48 h of treatment.

In this context, the possibility that phenomena of senescence were triggered by common mallow extract was also verified by applying a β-galactosidase staining assay. The kit stains the senescent cells in dark green, allowing them to be counted by optical microscopy. As shown in the representative image reported in Figure 3D, in the HCT-116 cellular system, no stained cells could be detected under any treatment conditions for 48 h, including in the CNT. However, Caco-2 cells revealed green staining after exposure to MFE for 48 h. Only 5% of CNT cells appeared senescent, as expected considering that this process is spontaneous and natural in a cell population, while the treatments at 0.9, 6 and 15 mg/mL caused an increase in the percentage of green cells equal to +13%, +24% and +34%, respectively, compared to the control (Figure 3E).

**Figure 4:**
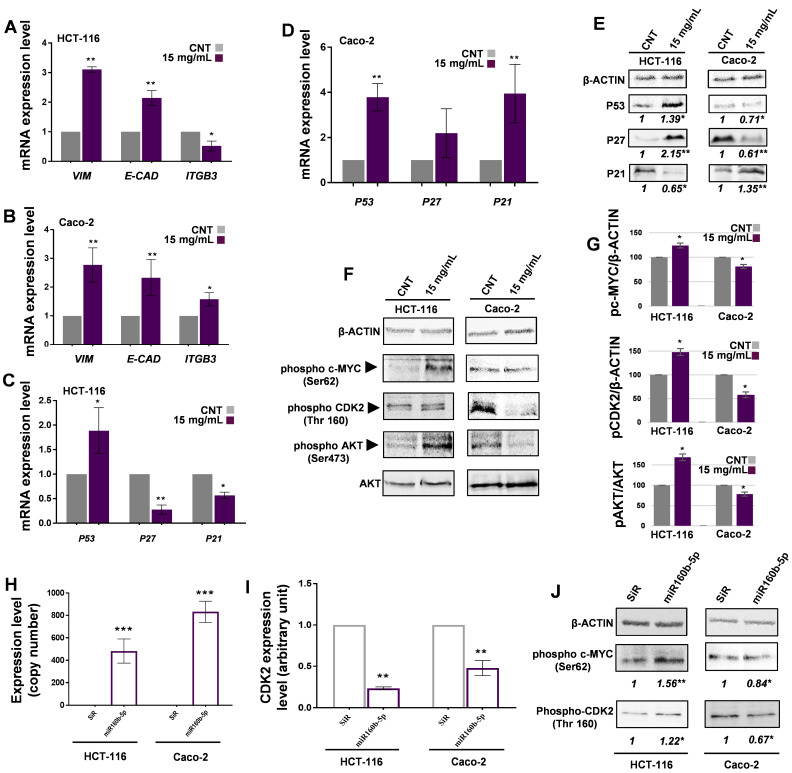
Molecular mechanisms activated by MFE in the tumour cells. (**A**–**D**) Results of RT-qPCR analyses carried out on the RNA isolated from HCT-116 (panels (**A**,**C**)) and Caco-2 (panels (**B**,**D**)) cells treated for 48 h with the highest dose of MFE. The data relative to *VIM*, *E-CAD*, *ITGB3* (panels (**A**,**B**)), *P53*, *P27* and *P21* (panels (**C**,**D**)) transcript levels were expressed as fold change compared to the respective internal CNT taken as unit (i.e., 1), after normalization for *β-ACTIN* mRNA according to the 2^−∆∆Ct^ formula. (**E**–**G**) Representative immunoblots of P53, P27, P21 (panel (**E**)), phospho c-MYC (Ser62), phospho CDK2 (Thr160), phospho AKT (Ser 473), AKT and β-ACTIN (panel (**F**)) protein levels in HCT-116 and Caco-2 cells, exposed or not for 48 h to the highest dose of MFE. Considering the protein signal of the CNT as a unit (i.e., 1), the quantitation of each band is reported immediately below its representative image in the panel (**E**). Results of the immunoblots presented in panel (**F**) are reported in the form of graphs in panel (**G**) as percentage variation compared to the CNT taken as unit (100%). β-ACTIN was used as a loading control to normalise the results. (**H**) The graph shows the transfection efficiency of the synthetic miR160b-5p after transfection in HCT-116 and Caco-2 cells for 48 h. (**I**) *CDK2* mRNA levels, measured by RT-qPCR, in HCT-116 and Caco-2 cells transfected for 48 h with synthetic miR160b-5p. Data were reported relative to CNT cells transfected with a scramble miRNA (SiR) and taken as unit (i.e., 1) after normalization for *β-ACTIN* mRNA, according to the 2^−∆∆Ct^ formula. (**J**) Representative immunoblots of phospho c-MYC (Ser62), phospho CDK2 (Thr160) and β-ACTIN protein levels in HCT-116 and Caco-2 cells transfected for 48 h with synthetic miR160b-5p. Considering the protein signal of the CNT as a unit (i.e., 1), the quantitation of each band was reported immediately below its representative image. β-ACTIN was used as a loading control for the normalisation. In all panels, data were indicated as mean ± s.d. of three independent experiments (* *p* < 0.05 vs. control; ** *p* < 0.01; *** *p* < 0.001).

**Common mallow extract modulates gene expression in a cell-type-dependent manner.** Epithelial–mesenchymal transition (EMT) plays a key role in colorectal cancer cell migration and invasion; for this reason, the level of some specific markers linked to this phenomenon were monitored. Since MFE exhibited its most significant activity at the highest dose, all subsequent experiments aimed at investigating the molecular mechanisms underlying the bioactivity of common mallow extract were conducted exclusively at this concentration (15 mg/mL). Thus, the expression of three genes codifying important adhesion proteins, that is *VIMENTIN* (*VIM*), *E-CADHERIN* (*E-CAD*) and αVβ3-*INTEGRIN* (*ITGB3*), were analysed by RT-qPCR (Figure 4A,B).

Results indicated that, after 48 h, MFE drastically increased the expression level of *VIM* mRNA in both cell lines: in particular, +2.05-fold in HCT-116 cells and +1.76-fold in Caco-2, compared to the respective control. In addition, in HCT-116, the content of *E-CAD* transcript was augmented by 1.14-fold, while that of *ITGB3* was reduced by 0.51-fold, with respect to the CNT. On the other hand, the same mRNAs increased by 1.32- and 0.57-fold, in that order, in Caco-2 cells, compared to the CNT sample. RT-qPCR analysis was also carried out to analyse the expression levels of the genes *P53*, *P27* and *P21* (Figure 4C,D), well-known to be involved in the modulation of cell fate. In both HCT-116 and Caco-2 cells, an increase in *P53* transcript was observed (+0.88- and +2.78-fold, respectively), compared to the CNTs. Unexpectedly, while *P27* and *P21* expression significantly decreased in HCT-116 (by 73% and 44%, respectively), the content of these mRNAs appeared upregulated in Caco-2 cells, although the variation in *P27* could not be considered reproducible (*p* > 0.05).

Lastly, CCNB1 (G2/mitotic-specific cyclin-B1) gene expression was also measured. The results revealed that mRNA significantly decreased by +0.42% in the treated Caco-2 cells, compared to the control, while it seemed to remain stable in HCT-116 exposed to MFE.

**MFE influences the level of proteins related to cell growth, differentiation and death.** Since the level of a transcript are not necessarily indicative of the content of the respective protein, due to the existence of post-transcriptional, translational and post-translational regulation levels, Western blot analyses were carried out on the protein extracts from the HCT-116 and Caco-2 cells. As shown in the representative blots and relative densitometric quantitation (Figure 4E), MFE caused in HCT-116 an upregulation of P53 and P27 protein content (+0.39- and +1.15-fold, respectively, compared to the CNT), while a decrease in P21 (−0.45-fold) was observed. By contrast, in Caco-2 cells, P53 and P27 protein levels diminished after exposure to MFE for 48 h (−0.29- and −0.39-fold, respectively), whereas an increase in P21 was observed (+0.35-fold) with respect to the control.

At this point, to understand how *M. sylvestris* could influence tumour cell fate, the concentrations of three proteins involved in the main molecular mechanisms modulating the cell cycle progression and transformation were investigated. The proteins were (i) CYCLIN-DEPENDENT KINASE 2 (CDK2); (ii) the proto-oncoprotein c-MYC (c-MYC); and (iii) the PROTEIN KINASE B (AKT). As evidenced in the representative blots reported in Figure 4F and relative quantitation shown in Figure 4G, after 48 h of incubation with the common mallow extract, HCT-116 cells presented an upregulation of the activated forms (i.e., phosphorylated) of CDK2 (Thr160) and c-MYC (Ser62) by 46% and 24%, respectively, compared to the CNT. Similarly, the level of phosphorylated AKT (Ser473) normalised on the total amount of AKT, increased by 75%. By contrast, in Caco-2 cells, MFE led to a decrease in phospho-CDK2 (−45%), phospho-c-MYC (−23%) and the ratio phospho-AKT/AKT (−26%), with respect to the CNT.

**Mallow flower miR160b-5p exerts a cross-kingdom effect on the tumour cell lines.** MFE has recently been characterized by Villani et al. [20], who documented its phenolic phytochemical and plant miRNA content, such as miR160b-5p. In view of the identification of *CDK2* mRNA as a predicted human target for miR160b-5p by Villani et al. [20], and considering the previously observed modulation of the CDK2 protein content by MFE, the subsequent objective was the quantification of *CDK2* transcript levels in treated cells via qPCR assays. Upon MFE exposure, a significant reduction was observed in HCT-116 (33%) and Caco-2 (37%) cells, compared to CNT (Supplemental Material—Figure S1). Based on these cumulative findings, the direct biological impact of synthetic miR160b-5p on HCT-116 and Caco-2 cell lines was investigated. To do this, the exogenous miR160b-5p was transfected in the tumour cells at the same concentration it was estimated in MFE (that is, 98 nM in a 15 mg/mL dose). After 48 h of incubation, the intracellular miRNA pool was purified from the human cells. Thus, the level of miR160b-5p was quantified by the RT-qPCR absolute method. As expected, the plant miRNA was detected only in transfected HCT-116 and Caco-2 cells, while no signal was found in the controls (with scrambled miRNA, SiR) (Figure 4H). By contrast, the human small RNA U6 was present in all samples, demonstrating the correctness of the procedure. Lastly, to further substantiate our work, both cell lines were treated with MFE to verify the presence of miR160b-5p within their cytoplasmic extracts. The detection of this signal confirmed the successful cellular uptake and internalization of this miRNA during the treatment process. At this point, the expression level of *CDK2* mRNA was measured in both miR-transfected cell lines, since, as stated above, the bioinformatics prediction indicated this transcript as a putative human target for the miR160b-5p. The results indicated that the synthetic miRNA strongly reduced the gene expression of *CDK2* in both cell lines (i.e., 77% in HCT-116 and 53% in Caco-2), compared to SiR controls (Figure 4I).

Lastly, in order to check if the downregulation of *CDK2* transcript, putatively induced by the exogenous miR160b-5p, was correlated to the same modifications of molecular signal transduction observed in Caco-2 and HCT-116 cells exposed to MFE, a Western blotting analysis was carried out. As shown in Figure 4J, compared to SiR samples, phospho-CDK2 and phospho-c-MYC levels accumulated in treated HCT-116 cells but diminished in Caco-2 cells, as occurred when these two cell lines were treated with the whole MFE.

## 4. Discussion

At least 35% of all cancers seem to be linked to an incorrect diet, and for colon tumour cases, this percentage would grow up to 80%. In addition, several studies have identified plant-like sources of potential antineoplastic agents, and it has been estimated that a diet rich in phytochemicals can reduce cancer onset, development and progression by 20% [30,31,32]. In fact, although these compounds play a key role in plants, in terms of environmental adaptation, communication and propagation mechanisms [33], it has been demonstrated that their effect may also be extended to the animal systems which come into contact with them, for instance, through diet [34,35]. For this reason, food consumers, pharmaceutical companies and scientists have increasingly focused their attention on the usefulness of plant-based products for the prevention and the potential treatment of pathological conditions, including inflammation, dysmetabolic stages and cancer [36,37].

To date, the World Health Organization (WHO) has estimated that about 65% of the global population depends on herbal medicine [38], while the Food and Drug Administration (FDA) and the European Medicines Agency (EMA) have declared that one quarter of all approved pharmaceutical compounds derive from plants [37,39,40,41]. This evidence shows that the plant kingdom represents a continuous source of bioactive metabolites, justifying the growing interest in traditional knowledge and folk medicinal practices recorded in recent decades in the search for new herbal drugs. For this reason, the main objective of the present contribution was to study the possible antineoplastic effect of *M. sylvestris*, an aspect under-investigated in the scientific literature. In fact, although this plant has an extensive ethnobotanical application in the Mediterranean area, especially for the treatment of gastrointestinal, hepatic and urinary inflammation [16], the molecular mechanisms underlying the effects of the common mallow in mammalian systems have not yet been clarified.

Recently, Villani et al. [20] have isolated several extracts from *M. sylvestris* leaves and flowers, using various solvents, and characterized their phytochemical and miRNA profiles. Based on these findings, the mallow flower extract (MFE) produced in that work was selected for the present study due to its superior richness and compositional variety. A key advantage of MFE lies in its final resuspension into an aqueous medium (as detailed in the Section 2). This process effectively eliminates any residual ethanol from the initial extraction steps, which is particularly significant given the known toxicity of alcohol in the human body. Furthermore, this aqueous formulation aligns with the traditional medicinal use of *M. sylvestris*, which is typically consumed as a decoction.

As already stated above, *M. sylvestris* is known to exert beneficial effects mainly on the gastrointestinal tract; thus, two human colorectal cell lines (i.e., HCT-116 and Caco-2) were chosen as in vitro model systems in the present study. In particular, among the types of cancer related to the gastrointestinal tract, these are the most lethal, aggressive and drug-resistant, affecting both men and women with the same incidence that continues to increase worldwide [42].

To obtain preliminary data about the potential cytotoxicity and antiproliferative effect of MFE, a Trypan blue exclusion test and MTT assay were carried out, in which cells were treated for 24 and 48 h with increasing concentrations of extract. In both cell lines, MFE induced a reduction in cell growth in a dose- and time-dependent manner, although the treatment did not determine a significant toxicity (Figure 1A–F). In parallel, to exclude the possibility that the mallow extract exerted negative effects on non-tumour cells, in view of a potential application of this extract in antineoplastic therapies, the same treatments and tests were also performed on immortalised human colon epithelial cells (HCEC1-ct), demonstrating that MFE could be safe for non-cancerous tissues (Supplemental Material—Table S2).

To date, it is not fully understood how phytochemicals act on human cells, or rather, each one of them seems to possess a different mechanism of action. However, are they always internalised? In what way? What are their direct targets? Are they able to activate specific membrane receptors, triggering peculiar intracellular signal transduction pathways? These are just some of the questions that remain difficult to answer clearly. Indeed, although some evidence has been collected, it seems that the bioactivity of each plant compound may be linked to various factors, like its chemical structure, bioavailability, concentration and cellular context [43,44,45,46,47]. For these reasons, to gain information about the molecular effects that MFE induced on our cancer models, the localisation of its phytochemicals in the treated cells was monitored by detecting the presence of these substances in the mammalian cells by fluorescent microscopy. The analysis showed that in both cell lines the plant secondary metabolites entered the cells and accumulated specifically in the nuclei (Figure 2A). The capability of phenolics, such as flavonoids, to bind DNA and to interact with various chromatin proteins has been ascertained; however, the exact binding sites, mode of interaction and function of phytochemicals on gene expression have not been fully clarified yet [48]. Regardless, the presence of MFE compounds in the cells clearly proved that these molecules were able to cross the cell membranes and may interfere with some nuclear targets.

The excessive production of reactive species, triggered by extracellular or intracellular stimuli, can cause damage to cell components, even leading to death. Thus, since MFE was found to be rich in antioxidants (e.g., phenolics), the variations in the cellular redox state after exposure to the plant extract were evaluated. The results suggested that an increase in reactive oxygen species (ROS) occurred only in Caco-2 cells after exposure for 48 h to the highest concentration of *M. sylvestris*, while minimal and non-significant changes were observed in HCT-116 (Figure 2B,C). This effect was not so unexpected; indeed, many studies have demonstrated that plant secondary metabolites are known to be excellent free radical scavengers, and they can also act as pro-oxidants, especially in in vitro conditions when used at high doses [49,50,51]. According to the literature, plant compounds, especially polyphenols, can in fact cause oxidative DNA breaks, produce new radical forms by interacting with transition metal ions (e.g., iron and copper), induce mitochondrial alterations (i.e., DNA mutations, respiratory chain damage, membrane permeability loss), inhibit specific genes, etc. [5,52,53,54].

It is noteworthy that, at low concentrations, reactive oxygen species (ROS) can function as critical intracellular messengers within signalling cascades, triggering the acquisition of various cancer hallmarks. Furthermore, ROS can be released into the tumour extracellular matrix, promoting inflammation and metastasis [55,56]. Consequently, our study proceeded to investigate processes typically associated with malignancy, such as migration and invasion, which may be indirectly modulated by fluctuations in ROS levels.

Wound assay and transwell tests documented how MFE reduced both the migration capability and the invasion power of Caco-2 and HCT-116. In fact, a strong inhibition of cell movement and penetration activity was observed in treated cells in a time- and dose-dependent manner (Figure 2D–J), suggesting a specific negative effect of common mallow extract on the metastatic potential of the investigated cancer cells.

Considering the previously described antiproliferative, antimigratory and antiinvasive effects exhibited by MFE, cell cycle analysis appeared mandatory at this point. Cytofluorimetric data demonstrated that the plant extract impaired the cell cycle of Caco-2, inducing an accumulation of cells in the G2/M phase. By contrast, no effect was observed for HCT-116 (Figure 3A,B).

Taken together, these findings indicate that MFE effectively inhibited both tumour cell growth and metastatic potential without inducing cell death. Evidence of the latter was supported by the Trypan blue exclusion test, which demonstrated the low cytotoxicity of the plant extract, and by the observation that the protein levels of several pro- and anti-apoptotic factors remained unchanged following treatment, confirming the absence of apoptotic pathway activation (Figure 3C). Thus, excluding this phenomenon, another hypothesis to be tested was represented by the stimulation of senescence. Senescence is an irreversible biological process that leads to cell metabolism slowdown and cycle arrest at the G0/G1 or G2/M phases [57]. Applying a kit able to detect β-galactosidase activity, a key enzyme for senescent cells [58], the capability of MFE to induce senescence in Caco-2 but not in HCT-116 was demonstrated (Figure 3D,E).

All the data collected until now were fascinating as they suggested that the two cell lines used in the present study reacted in a different way to the same plant extract, only sharing the effect of reduced proliferation. To further corroborate this aspect and shed light on the exact mechanism of action promoted by MFE in tumour cells, the expression rate of the main genes involved in cell growth and metastatization (i.e., *VIM*, *E-CAD*, *ITGB3*, *P53*, *P21*, *P27* and *CCN1B*) was monitored by RT-qPCR analysis (Figure 4A–D).

Surprisingly, HCT-116 and Caco-2 cells incubated with the highest dose of extract for 48 h showed a significant upregulation of the *VIM* transcript level. As reported in the literature, this gene is greatly involved in cell motility, proliferation, differentiation and apoptosis, although it may also act like a scaffold component for actin functions during mitosis [59,60]. However, unlike in other cases, in gastrointestinal tumours, an accumulation of this factor has been correlated to a decrease in proliferation and invasive potential [61,62], as documented in the present research. In both cell lines, the accumulation of *E-CAD* mRNA, a canonical signature of epithelial-like cells, supported the theory that the common mallow extract inhibited the EMT process [63]. Additional evidence was related to *ITGB3*; the expression of this gene was repressed by MFE in HCT-116 cells, as is expected for cells that reduce their invasive and metastatic power [64,65], while in Caco-2 it significantly increased. This finding is in line with the results discussed above; in fact, this peculiar integrin had been identified in the literature as a typical marker of senescence [66,67]. To conclude, the level of *CCN1B* mRNA tended to remain stable in HCT-116 cells, whereas a decrease in its transcript was found in Caco-2. These data were consistent with the previous cell cycle analysis, as CCN1B is required for G2/M transition, which appeared inhibited in Caco-2 cells [68].

Regarding the *P53*, *P27*, and *P21* genes, a different trend was observed for the two cell types, as well as between their respective mRNA and protein levels (Figure 4C–E). The former observation was expected, given that HCT-116 and Caco-2 exhibited distinct responses to MFE treatment, while the latter discrepancy could be attributed to the well-known lack of correlation between transcriptional and translational rates, in addition to the varying stabilities of transcripts and proteins. The differential response of the two cell lines was most likely associated with their distinct P53 status. In fact, HCT-116 expresses wild-type P53, whereas Caco-2 cells are P53-null, or rather harbour mutant/deleted P53 alleles, resulting in non-functional protein isoforms [69,70]. Therefore, comparing these two cell models was crucial for elucidating how human colon cancer cells modulate their biological response to MFE in the presence or absence of functional P53, providing deeper insights into the role of this *guardian of the genome* in tumour biology.

The *P53*, *P27* and *P21* genes increased their expression in Caco-2 cells. By contrast, in HCT-116, only the level of *P53* mRNA appeared upregulated, while that of the *P21* and *P27* genes was reduced. As stated before, *P53* is a gene notoriously involved in cancer; thus, the induction of its expression in both cell lines could be an indicator of activation of control mechanisms reducing cell proliferation in response to MFE, although recent studies have shown that it may also participate in the induction of senescence [71,72,73,74].

Despite the observed upregulation of P53 transcript, the lack of accumulation of the respective protein in Caco-2 could be explained by the instability of its deleted form. In this case, since exogenous treatments often act as cellular stressors that trigger P53-mediated signalling cascades, the absence of an active P53 would have triggered alternative compensatory pathways. Indeed, the increased level of the P21 protein in this cell line was perfectly in line with the cell cycle arrest and senescence induction documented above [75,76,77]. In particular, it is important to underline that the literature has stated that ROS-induced senescence is specifically promoted by P21, whose activation (for instance by SP1 factor) would be P53-independent in this P53-null cell line [78,79,80]. This link is coherent with the data collected in the current work on Caco-2 cells, where a simultaneous increase in ROS, P21 and β-galactosidase was detected.

In HCT-116, Western blotting analyses revealed an accumulation of both P53 and P27. In this context, MFE potentially stimulated P53 production, which in turn stabilized P27, explaining its accumulation even when its gene expression rate was low. More in detail, a high level of P53 would indirectly inhibit SCF-SKP2, an E3 ubiquitin ligase responsible for the degradation of P27 [81,82]. Thus, the latter, in its cytoplasmatic form, might work as an anti-apoptotic factor [83,84]. This condition, in combination with low levels of P21, could be considered a premise for differentiation events [85,86]. Indeed, HCT-116 showed a more elongated shape after exposure to the plant extract, suggesting that *M. sylvestris* may have differentiative properties on this cell line.

In this study, three other protein targets were analysed to clarify the molecular mechanism underlying the bioactivity of *M. sylvestris* extract on HCT-116 and Caco-2 cells: CDK2, c-MYC and AKT (Figure 4F,G). These factors were selected because they are also regulators of cell growth, proliferation and even senescence [87,88,89]. Specifically, CDK2 is a key factor for the transition from the G2 to the M phase, c-MYC is a target of CDK2, while Akt is involved in the activation of the senescence process and cell cycle progression [90,91]. Particular attention was initially paid to CDK2, taking into account that MFE seemed to determine a G2/M phase arrest in Caco-2 cells, as demonstrated by cytofluorimetric analysis. Thus, the content of the phosphorylated and active forms of CDK2 (Thr160) and c-MYC (Ser62) was detected by specific antibodies. The interest regarding these two proteins was also due to the fact that they both are inhibitors of senescence [92], another phenomenon documented in Caco-2 cells exposed to the plant extract. Western blotting experiments indicated that in HCT-116-treated cells, both proteins tended to be upregulated. This was consistent with the absence of senescence induction in this cell line after MFE exposure, as c-MYC is known to promote escape from senescence. In particular, the ability of c-MYC to suppress senescence is dependent on its phosphorylation at Ser62 residue (the phosphorylated site monitored here), which is performed precisely by CDK2 [92]. In parallel, the increase in phospho-AKT levels detected in this cell line was also consistent with the trend of the CDK2/c-MYC axis [93,94]. Consistent with its role as a primary repressor of *P21*, the upregulation of c-MYC likely contributed to the concomitant reduction in the levels of this protein. Remarkably, this inverse correlation persisted even as P53 content showed a tendency to increase [95]. Indeed, the observed upregulation of CDK2 might be attributed to the concomitant downregulation of its inhibitor, P21 [96]. On the other hand, the lack of a repressive effect on CDK2 by the increased levels of P27 might be linked to the fact that this function is strictly dependent on nuclear localization. Thus, we hypothesize that the high levels of AKT observed in our model may lead to P27 phosphorylation (potentially at Thr157 or Thr198), triggering its export from the nucleus to the cytoplasm [97]. In this compartment, rather than regulating CDK2, P27 might instead exert anti-apoptotic effects and promote cellular differentiation, as already stated above, which explains why its increase did not correlate with CDK2 inhibition.

By contrast, in Caco-2 cells, a downregulation of phospho-CDK2 and phospho-c-MYC protein content was observed, allowing P21 to trigger senescence [98]. Moreover, the reduction in phospho-AKT level corroborated the premature senescence induction in these cells, as already stated in the literature [93,94,99]. In this cellular context, the observed downregulation of CDK2 appeared to be primarily driven by a robust induction of P21 by ROS, despite a concomitant reduction in P27 levels, which may be attributed to protein instability resulting from physiologically low P53 content [81,82].

As stated at the beginning of the discussion, the *M. sylvestris* flower seems to be characterized by eight typifying miRNAs, according to the miRNome analysis carried out by Villani and colleagues [20]. Among them, miR160b-5p appeared very interesting because the bioinformatic analysis carried out by the same authors has suggested that one of the most valid human targets for this short nucleic acid could be the CDK2 transcript. In fact, a downregulation of the *CDK2* transcript level was registered in the present work, in HCT-116 and Caco-2 cells exposed to MFE, corroborating the prediction. Therefore, the possibility that miRNAs contained in the *M. sylvestris* flower contributed to the biological effect of the plant extract on the cancer cells was explored. In particular, the fact that cross-kingdom regulation of HCT-116 and Caco-2 gene expression could be exerted by the plant miR160b-5p was investigated (Figure 4H–J), as done in the past for another plant miR [11].

At this point, the presence of miR160b-5p in MFE was checked, its concentration was measured and its internalisation capability in HCT-116 and Caco-2 was assessed. Then, both cell lines were transfected with a synthetic miRNA, which mimicked miR160b-5p, at the same concentration found in MFE. The presence of the exogenous miRNA was detected exclusively in transfected cells, which were consequently characterized by a significant downregulation of *CDK2* mRNA levels. These data showed that miR160b-5p was internalized into the cells and interfered with *CDK2* gene expression, as predicted in Villani et al. [20]. However, the two cell lines responded differently to the treatment with the synthetic miRNA; in HCT-116, phospho-c-MYC and phospho-CDK2 protein content appeared upregulated, while in Caco-2 they both reduced. These trends were similar to those observed in the two cell lines when exposed to the whole MFE. Thus, while these results in Caco-2 cells can easily be explained by the fact that the *CDK2* gene was silenced by miR160b-5p, the situation in HCT-116 appeared more complicated. In this case, a compensatory mechanism may have been activated by the cells to maintain high levels of CDK2 protein and its target, c-MYC. A specific genetic set (e.g., mutations in key genes) and a peculiar physiological/metabolic pattern may be responsible for this type of response of HCT-116 cells to miR treatment. Surely, this is preliminary evidence that needs to be further validated, but it suggests that the effect of MFE on the tumour cells may result from its miRNA content and not, or not only, from its phytochemicals.

## 5. Conclusions

**Figure 5:**
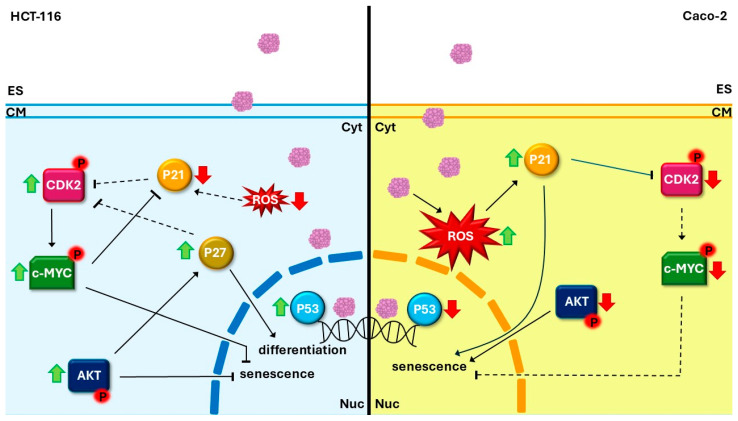
Hypothetical model of MFE action in tumour cells. The possible signalling activated in HCT-116 (on the left) and Caco-2 (on the right) cells treated with MFE (indicated with light violet spots) is shown. In the HCT-116 cells, a block of senescence mediated by an upregulation of the CDK2/c-MYC/AKT axis and a hypothetic differentiation event modulated by cytoplasmatic P27 is outlined. On the other hand, in Caco-2 cells, the activation of senescence mediated by an increase in P21, a reduction in AKT and an accumulation of intracellular ROS levels is represented. Legend—green up arrows indicate an increase in the related components inside the cell; red down arrows indicate a decrease in the related components inside the cell; continuous black arrows indicate that the signal is active; dashed black arrows indicate that the signal is not active; black arrows with tips indicate a promoting activity; black arrows without tips indicate inhibitory activity. Abbreviations—Cyt: cytoplasm; CM: cell membrane; ES: extracellular space; Nuc: nucleus; P: phosphorylated protein; ROS: reactive oxygen species.

According to Mediterranean folk medicine, *M. sylvestris* seems to be highly recommended for the treatment of gastrointestinal disorders. Consequently, this research explored the antineoplastic properties of this species across two colorectal cancer models (HCT-116 and Caco-2) to identify the specific molecular pathways activated by its phytochemical components. The extract obtained from the flower of this species (MFE) showed antiproliferative, antimigratory and antiinvasive effects on both cell lines. However, while the treatment was able to induce senescence in Caco-2, HCT-116 exposed to MFE appeared to differentiate, suggesting a cell-type-dependent effect of the plant extract. A possible mechanism of action of MFE on HCT-116 and Caco-2 cells, based on the data obtained in this work, was summarised and presented in Figure 5. Lastly, the capability of a miRNA detectable in MFE (i.e., miR160b-5p) to carry out cross-kingdom regulation of the gene expression of the two cell lines was demonstrated, suggesting that not only phytochemicals but also these short nucleic acids can participate in determining the bioactivity of the plant extract. All these data provide information which could support the development of new food supplements and drugs based on *M. sylvestris* flowers. Certainly, the necessity to translate this preliminary evidence into in vivo studies is mandatory in order to confirm the results obtained here and to propose the possible use of this medicinal plant for therapeutic purposes. Finally, the last part of the work contributes to opening new perspectives in the field of gene therapy based on plant miRNAs and extracts.

## Data Availability

The information related to the research is reported in the main text or in the Supplementary Materials of the manuscript. Vouchers of the plant material are preserved in the Laboratory of Botany at the University of Rome Tor Vergata.

## Supplementary Materials

The following supporting information can be downloaded at https://www.mdpi.com/article/10.3390/nu18030495/s1, Table S1: Primer sequences used for RT-qPCR analysis and relative data [100,101,102,103,104]; Table S2: Mean raw data obtained from the counting assays for HCT-116, Caco-2 and HCEC-1CT cell lines; Figure S1: *CDK2* expression level in HCT-116 and Caco-2 cells exposed to MFE (15 mg/mL), compared to CNT conditions.

## Abbreviations

DCFH-DA	2′,7′-dichlorodihydrofluorescein diacetate
DPBA	Diphenylboric acid 2-aminoethyl ester
MTT	3-(4,5-dimethyl-thiazol- 2-yl)-2,5-diphenyltetrazolium bromide kit
CASP-3	Caspase-3
cCASP-3	Cleaved Caspase-3
CNT	Control
CDK2	Cyclin-dependent kinase 2
DMEM	Dulbecco’s Modified Eagle’s Medium
E-CAD	E-Cadherin
FBS	Fetal bovine serum
CCN1B	G2/mitotic-specific cyclin-B1
HCT-116	Human colon cancer cell line
HCEC-1CT	Human colon epithelial cell line
Caco-2	Human colorectal adenocarcinoma cell line
MFE	Mallow flower extract
miRNAs, miRs	MicroRNAs
ROS	Reactive oxygen species
PBS	Phosphate-buffered saline
AKT	Protein kinase B
c-MYC	Protooncoprotein cMyc
SiR	Scrambled miRNA
VIM	Vimentin
ITGB3	αVβ3-Integrin
