# Supplementary material for: Malva sylvestris Flower Extract Exhibits Antineoplastic Potential Against Human Colon Cancer Cell Lines and Induces CDK2 Transcript Instability via Plant miR160-5p
Source: Nutrients. 2026 Feb 2;18(3):495. doi: 10.3390/nu18030495 (PMC12899310; doi:10.3390/nu18030495)

Supplemental Material – Table S1. Primer sequences used for RT-qPCR analysis and relative data.

**Acronym**	**Gene name – gene function – NCBI gene ID**	**Sequences**	**References***	**Basal Ct in HCT-116 CNT cells**	**Basal Ct in Caco-2 CNT cells**
VIM	*VIMENTIN* - Gene codifying for a major intermediate filament protein – ID: 7431.	F: 5′-GACAATGCGTCTCTGGCACGTCTT-3′<br>R: 5′-TCCTCCGCCTCCTGCAGGTTCTT-3′	Pan et al., 2012	25.7	25.4
E-CAD	*E-CADHERIN* – Gene codifying a key cell-cell adhesion molecule – ID: 999.	F: 5’-TGGAGGAATTCTTGCTTTGC-3’<br>R: 5’-CGTACATGTCAGCCAGCTTC-3’	Vetrini et al., 2004	24.5	23.9
ITGB3	*INTEGRIN αVβ3* – Gene codifying for a cell surface receptor involved in cell adhesion and signalling – ID: 3690.	F: 5′-GACTGTGTGGAAGACAATGTCTGTAAACCC-3′<br>R: 5′-CCAGCTAAGAGTTGAGTTCCAGCC-3′	Pan et al., 2012	28.1	28.3
P53	*TUMOR PROTEIN P53* – Gene codifying for a protein that coordinates cellular stress responses – ID: 7157.	F: 5’- GTGAGCGCTTCGAGATGTTC-3’<br>R: 5’- CCCTTCTGTCTTGAACATGAG-3’	Zhang et al., 2017	28.1	33.6
P27	*CYCLIN-DEPENDENT KINASE INHIBITOR 1B* – Gene codifying for the protein p27Kip1 interacting with CDK/Cyclin complexes – ID: 1027.	F: 5’- ATAAGGAAGCGACCTGCAAC-3’<br>R: 5’- ACGTTTGACGTCTTCTGAGG-3’	Zhang et al., 2017	27.5	27.1
P21	*CYCLIN-DEPENDENT KINASE INHIBITOR 1A* – Gene codifying for the protein p21WAF1/Cip1 interacting with CDK/Cyclin complexes – ID: 1026.	F: 5’- AGACCAGCATGACAGATTTC-3’<br>R: 5’- ACTGAGACTAAGGCAGAAGA-3’	Zhang et al., 2017	28.2	29.4
CCNB1	*CYCLIN B1* - Gene codifying for a protein essential for cell cycle progression – ID: 891.	F: 5’- AGACCAGCATGACAGATTTC-3’<br>R: 5’- ACTGAGACTAAGGCAGAAGA-3’	Lin et al., 2017	23.2	23.5
CDK2	*CYCLIN-DEPENDENT KINASE 2* - Gene codifying for a kinase critical for the G1/S transition and S-phase progression – ID: 1017.	F: 5’- GGAAACCAGGAAGCCTAGCATC-3’<br>R: 5’- GGATGATTCAGTGCCATTTTGCC-3’	Lin et al., 2017	22.9	23.2
β-ACTIN	*β-ACTIN* – Gene codifying a highly conserved structural protein (used as loading control) – ID: 60.	F: 5’-ACCACCATGTACCCTGGCATT-3’<br>R: 5’-CCACACGGAGTACTTGCGCTCA-3’	Simard and Chabot, 2000	22.2	22.2

*References are present in full version in the bibliography of the paper.

Supplemental Material – Table S2. Raw data used for the production of graphs relative to counting assays for HCT-116, Caco-2 and HCEC-1CT cell lines.

**HCT-116**	Trypan blue staining (viable cell number)			Trypan blue staining (non viable cells %)		MTT assay (relative cell number %)	
	0 h	24 h	48 h	24 h	48 h	24 h	48 h
CNT	150000*	425000± 17000	1125000± 56300	0.5±0.1	0.5±0.25	100*	100*
0.9 mg/mL	150000*	240000± 14400	720000± 21000	4.5±1.5	2.5±1.0	81±4	75±6
6 mg/mL	150000*	205000± 10200	550000± 22000	7.5±1.7	6.5±0.2	78±4	51±4
15 mg/mL	150000*	180000± 6300	528000± 20720	12.0±4.0	10.5±0.25	69±2	56±2

*Starting point with no standard deviation.

**Caco-2**	Trypan blue staining (viable cell number)			Trypan blue staining (non viable cells %)		MTT assay (relative cell number %)	
	0 h	24 h	48 h	24 h	48 h	24 h	48 h
CNT	150000*	385000± 15400	1190000± 170000	2.0±1.0	1.8±0.2	100*	100*
0.9 mg/mL	150000*	245000± 12250	1095000± 43000	3.0±2.0	2.0±0.1	94±5	73±5
6 mg/mL	150000*	175000± 10500	935000± 102000	10.5±0.5	2.5±1.3	92±2	72±6
15 mg/mL	150000*	135000± 6750	595000± 90000	15.5±2.0	7.5±2.3	90±5	47±5

*Starting point with no standard deviation.

**HCEC-1CT**	Trypan blue staining (viable cell number)			Trypan blue staining (non viable cells %)		MTT assay (relative cell number %)	
	0 h	24 h	48 h	24 h	48 h	24 h	48 h
CNT	150000*	320000± 18000	850000± 51000	0.5±0.1	0.5±0.1	100*	100*
0.9 mg/mL	150000*	325000± 15000	855000± 48000	1.0±0.1	1.5±0.2	98±3	101±2
6 mg/mL	150000*	315000± 14000	865000± 53000	2.5±0.5	2.0±0.5	98±4	97±3
15 mg/mL	150000*	290000± 15500	840000± 47000	3.5±0.5	4.5±1.0	96±3	97±2

*Starting point with no standard deviation.

Supplemental Material – Figure S1. *CDK2* gene expression level in HCT-116 and Caco-2 cells exposed to MFE (15 mg/mL), compared to CNT conditions (**$p<0.01$).

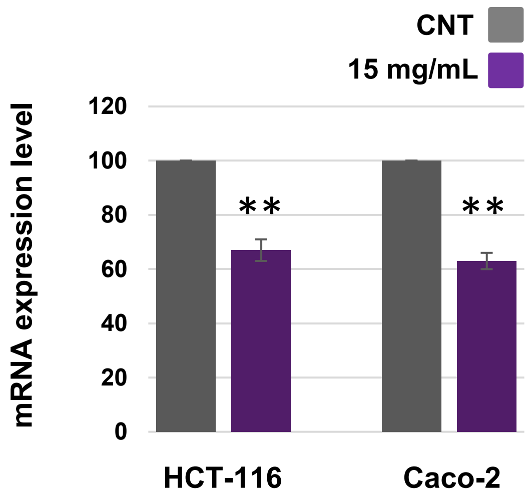

Supplement: Supplementary file 1 [file nutrients-18-00495-s001.zip › nutrients-4092699-supplementary.pdf]
